# Crystal structures of FMN‐bound and FMN‐free forms of dihydroorotate dehydrogenase from *Trypanosoma brucei*

**DOI:** 10.1002/2211-5463.12403

**Published:** 2018-03-06

**Authors:** Tomomi Kubota, Osamu Tani, Tomohiko Yamaguchi, Ichiji Namatame, Hitoshi Sakashita, Koji Furukawa, Kazuhiko Yamasaki

**Affiliations:** ^1^ Biomedical Research Institute National Institute of Advanced Industrial Science and Technology (AIST) Tsukuba Japan; ^2^ Drug Discovery Research Astellas Pharma Inc. Tsukuba Japan

**Keywords:** apoform, conformational flexibility, flavin‐binding enzyme, neglected tropical disease, peptide flipping, *Trypanosoma brucei*, X‐ray crystallography

## Abstract

Dihydroorotate dehydrogenase (DHODH) is a flavin‐binding enzyme essential for pyrimidine biosynthesis, which converts dihydroorotate to orotate. Three‐dimensional structures of cytosolic DHODH of parasitic protozoa are of interest in drug discovery for neglected tropical diseases, especially because these enzymes possess significantly different structural and functional properties from the membrane‐associated human enzyme. The existing crystal structures of the flavin mononucleotide (FMN)‐bound DHODHs reveal a number of interactions stabilizing FMN. However, to understand the binding mechanism correctly, it is necessary to compare the structures of the FMN‐bound and FMN‐free forms, because the protein moiety of the former is not necessarily the same as the latter. Here, we prepared the FMN‐free DHODH of *Trypanosoma brucei* using an *Escherichia coli* overexpression system. Although this apoform lacks enzymatic activity, simple incubation with FMN activated the enzyme. It was stable enough to be crystallized, enabling us to determine its structure by X‐ray crystallography at 1.6 Å resolution. We also determined the FMN‐bound form at 1.8 Å resolution. Although the two structures have essentially the same scaffold, we observed flipping of a peptide‐bond plane in the vicinity of the FMN‐binding site, accompanied by an alternative hydrogen‐bonding pattern. Comparisons of B factors of the protein main chain revealed that binding of FMN decreased flexibility of most of the residues at the FMN‐binding site, but increased flexibility of a lid‐like loop structure over the active center. This increase was ascribed to a conformational change in an FMN‐contacting residue, Asn195, which induced a rearrangement of a hydrogen‐bond network of the residues comprising the lid.

**Abbreviations**
DHODH: dihydroorotate dehydrogenase
FMN: flavin mononucleotide
ITC: isothermal titration calorimetry

Biosynthetic pathway of pyrimidine is an essential process in cell growth, which is one of the notable drug targets for extremely prolific cells, such as tumor and infected microorganisms 1, 2, 3, 4, 5. Dihydroorotate dehydrogenase (DHODH; http://www.chem.qmul.ac.uk/iubmb/enzyme/EC1/3/98/1.html for family 1A) is a key component of *de novo* pyrimidine synthesis and catalyzes oxidation of dihydroorotate to form orotate 6. DHODHs from a variety of organisms are classified into two subfamilies. Enzymes localized in cytosol, found in fungi and protozoa, are categorized into family 1 7. Family 1 is further divided into families 1A, 1B, and 1S. Family 1A enzymes bind flavin mononucleotide (FMN) as a redox cofactor, functioning as the hydrogen carrier, and utilize some organic acids such as fumarate as final hydrogen acceptors, while family 1B enzymes have flavin adenine dinucleotide and/or Fe–S cluster, in addition to FMN, and utilize nicotinamide adenine dinucleotide as the acceptor 8, 9, 10. A different type of DHODH, family 1S, was found from a thermoacidophilic archaeon *Sulfolobus solfataricus*, which has FMN and Fe–S cluster but cannot utilize NAD or fumarate 11. The enzymes found in multicellular organisms and Gram‐negative bacteria are categorized into family 2, which are anchored into the membranes, and utilize ubiquinone as the final acceptor 12.

Chagas’ disease and African sleeping sickness are among the most serious neglected tropical diseases, which are caused by parasitic protozoan trypanosomes and are termed Trypanosomiasis 13, 14, 15. Although more than a billion of people are at risk of infection, drug development for the diseases remains insufficient. Trypanosoma DHODHs, categorized into family 1A, are phylogenetically and functionally distant from the human enzyme, a family 2 enzyme 7. Inhibitors specifically tuned up against Trypanosoma enzymes are, therefore, unlikely to work against human enzyme; DHODH is considered to be a suitable target for antiprotozoan drug possible to dose humans 16. Orotate analogues which discriminate between human and protozoan enzymes to avoid harmful side effects were recently reported 17, 18.

Several crystal structures of DHODH belonging to family 1A from protozoa *Leishmania major* 19, *Trypanosoma brucei* 20, *Trypanosoma cruzi* 21, and *Leishmania braziliensis* 22 have been reported, which share the common folding scaffold. Inaoka *et al*. 21 determined the crystal structures of orotate‐, dihydroorotate‐, and fumarate‐bound forms of *T. cruzi* enzyme, enabling them to propose a catalytic mechanism and to identify active‐site residues. It was also revealed that pathway to the active center on the isoalloxazine ring of FMN is covered by a lid‐like structure composed of a flexible loop from Cys131 to Pro141 19. Dynamic features of the lid probably affect binding‐and‐release of substrates and products by a ping‐pong type mechanism 23. After binding of substrate, a catalytic residue, Cys131, located at the root of the lid, could be fixed at a proper position for catalysis concomitantly with closing of the lid. This control could rationally be affected by the redox state of FMN, as reported by Luo *et al*. 24 on proline dehydrogenase. Therefore, influence of FMN on the conformation of protein part is an important subject to study.

The above crystal structures provide clues to understand mechanism of FMN binding, by revealing a number of stabilizing interactions, such as hydrogen bonds. To correctly understand the mechanism, however, especially in terms of energetics, we should compare the structures of the FMN‐free and FMN‐bound forms, because the former is not necessarily the same as the protein moiety of the latter. Structural analyses of the FMN‐free form of a few other FMN‐binding proteins have been reported 22, 23, 24, 25, 26. Crystal structures of the FMN‐free form of flavodoxin, a redox FMN‐containing protein, from plant and *Helicobacter pylori* showed an induced fit‐type conformational change in the 50s loop and some residues around the FMN‐binding site 25, 26. Consistently, an NMR analysis on *Escherichia coli* flavodoxin indicated that several loops around the FMN‐binding site became highly mobile in the FMN‐free form 27. In addition, conformational changes in residues distant from the FMN‐binding site also contribute to a tight affinity with FMN, as revealed by NMR analyses on *Azotoboctor vinelandii* flavodoxin 28. The crystal structures of dibenzothiophene monooxygenase, DszC from *Rhodococcus erythropolis* 29, and a mutant of aromatic acid decarboxylase, UbiX, from a psychrophilic bacterium *Colwellia psychrerythraea* 30 indicated a conformational change between the FMN‐free and bound forms around the substrate‐binding site. Therefore, it is of considerable interest whether any conformational change takes place upon binding of FMN to DHODH or not.

In this study, we isolated the FMN‐free form from an enzyme source of *T. brucei* DHODH prepared by an *E. coli* expression system. The inactive FMN‐free form possessed the ability to convert to the FMN‐bound form that exhibited enzymatic activity. The crystal structure of the FMN‐free form has the similar protein scaffold to that of the FMN‐bound form, while a flipping of the peptide plane near the FMN‐binding site and a decrease in B factor of the lid structure were observed. These facts suggested that the conformational change in the FMN‐binding site affects opening and closing of the lid. The FMN‐free form was also utilized to determine the dissociation constant of FMN by isothermal titration calorimetry (ITC).

## Results

### Isolation of FMN‐free form

**Figure 1:**
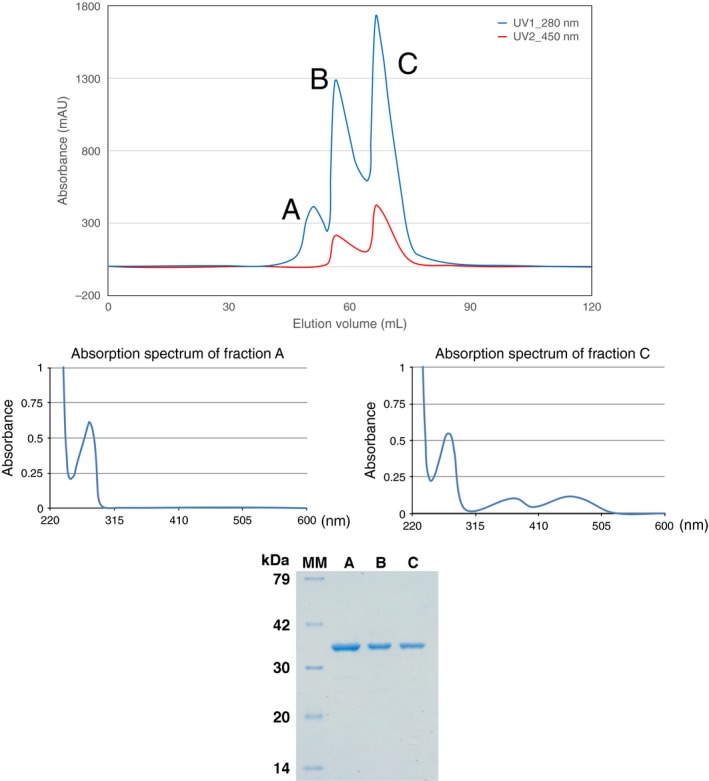
Separation of the FMN‐free and FMN‐bound DHODH with an ionic exchange chromatography. Sample was applied to RESOURCE Q (GE Healthcare) equilibrated at 20 mm Tris/HCl pH 8.0 and was eluted with a gradient of 0–200 mm NaCl for 20 column volumes, to completely separate the FMN‐free form from the FMN‐bound form. Elution pattern (upper panel) was monitored with absorption at 450 nm and 280 nm. Fractions A, B, and C were distinguished based on absorption ratio at the two wavelengths. Absorption spectra of fractions A and C are given in the middle panels. An SDS/PAGE analysis of the three factions is presented in the bottom, where molecular weight marker (Middle Range; Wako) was used.

*Trypanosoma brucei* DHODH was highly expressed in *E. coli*; expression level reached more than 60 mg proteins from 1‐L culture, which is likely to have caused a shortage of FMN in *E. coli* cell. Consequently, protein was divided into three fractions on an anion‐exchange chromatography: fraction A without visible absorption at 450 nm, fraction B exhibiting absorption at 450 nm, and fraction C with 450 nm/280 nm absorption ratio of approximately twice as large as fraction B (Fig. 1). It is clearer in the absorption spectra that fraction A has no absorption in the visible range, while fraction C exhibited that of the typical FMN‐bound enzyme as reported 31, 32 (middle panels in Fig. 1). Fraction B with a lower visible absorption is likely to contain a heterodimer composed of the FMN‐free and FMN‐bound forms, because both showed dimeric crystal structures (see below). Also, we confirmed that the three fractions contain the protein of the same size (bottom panel in Fig. 1).

We stocked fraction A as the solution containing the FMN‐free form of DHODH dimer and tested whether it regains an enzymatic function after reconstitution with FMN. Fraction A as was prepared did exhibit a very low dehydration activity (0.11 μmol·min^−1^·(mg protein)^−1^, kinetic data not shown). By incubation with FMN for only a few minutes prior to assay, this fraction gained activity of 18 μmol·min^−1^·(mg protein)^−1^, which was as high as 75% of fraction C, 26 μmol·min^−1^·(mg protein)^−1^. These values correspond to apparent *k*_cat_ of 10–15 s^−1^, which are comparable to a reported value, 8.5 s^−1^ 20. These results established that fraction A was the solution of the FMN‐free apoform.

**Figure 2:**
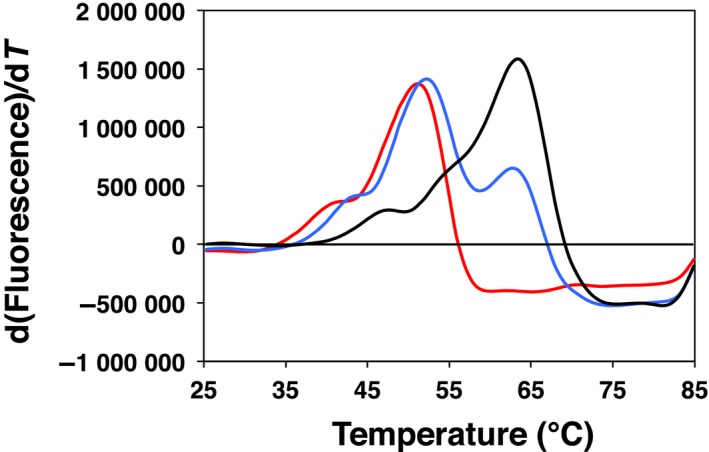
Fluorescence‐based thermal denaturation assay of TbDHODH in the FMN‐bound (black), FMN‐free (red), and heterodimeric (blue) forms. These correspond to fractions C, A, and B in Fig. 1, respectively. Derivative melting profiles are presented so that the peak centers correspond to the melting midpoints.

We evaluated stabilities of these forms by fluorescence‐based thermal denaturation assay (Fig. 2). Melting temperatures were 63.4 ± 0.0 and 51.2 ± 0.2 °C for the FMN‐bound and FMN‐free forms, respectively. Therefore, it is obvious that FMN significantly stabilizes the protein structure. In addition, the putative heterodimeric form (fraction B in Fig. 1) showed a mixed profile with two melting temperatures of 52.1 ± 0.2 and 62.7 ± 0.2 °C, which are nearly identical to those of the FMN‐free and FMN‐bound forms, respectively. This immediately indicates that a monomer in the dimeric DHODH denatures independently from the other monomer. Because association of denatured and folded proteins is unlikely, we suggest a possibility that a DHODH dimer dissociates into two monomers at higher temperatures (presumably above 40–47 °C, where we see shoulders in the profiles).

### Crystal structures of FMN‐free and FMN‐bound forms of DHODH

**Figure 3:**
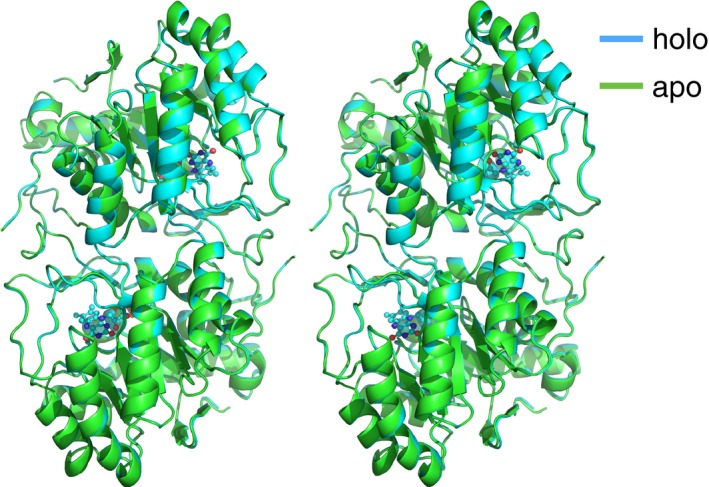
Stereo view of dimeric TbDHODH. The FMN‐free form in green (PDB: 5XFW, chains A and B) and the FMN‐bound form in cyan (PDB: 5XFV, chains A and B) are superimposed. Ball‐and‐stick model represents FMN.

Both the FMN‐free and FMN‐bound forms were crystallized under the similar conditions in the presence of malonate and citrate as crystallizing reagents. The crystals were isomorphous with each other. Their structures were determined with the molecular replacement method by use of a model, DHODH from *T. brucei* (PDB code: 2B4G) 20, at resolutions of 1.6 and 1.8 Å for the FMN‐free and FMN‐bound forms, respectively (Table 1). The dimeric features and the whole folding structures of the FMN‐free and bound forms were similar to each other (Fig. 3), indicating that no remarkable conformational change in the main‐chain folding took place upon binding of FMN.

**Table 1:** Crystal data, data collection, and refinement statistics

Crystal	FMN‐bound form (PDB: 5XFV)	FMN‐free form (PDB: 5XFW)
Cell		
Space group	*P2*_*1*_2_*1*_2	*P2*_*1*_2_*1*_2
Dimension *a*,* b*,* c* (Å)	139.9, 147.0, 66.5	140.1, 146.2, 66.4
No. of molecules in ASU	4 (2 dimers)	4 (2 dimers)
V_M_* (Å^3^Da^−1^)	2.32	2.33
Data collection		
Resolution (outer shell)	1.80 (1.83–1.80)	1.6 (1.63–1.60)
Completeness (outer shell)	99.0 (98.5)	99.4 (97.5)
Mean I/sigma (outer shell)	34.5 (7.1)	33.7 (2.5)
R‐merge (outer shell)	0.077 (0.206)	0.096 (0.98)
Wavelength	1.0000	1.0000
No. of reflections	1 431 499	2 405 603
No. of unique ref.	128.274	180 934
Refinement		
R‐work/R‐free	0.167/0.197	0.176/0.194
RMSD from ideal values		
Bond length (Å)	0.006	0.006
Bond angle (˚)	1.19	1.17
Rama plot (%)		
Favored	97.8	97.3
Allowed	2.2	2.7
Outlier	0	0
Average B factors (Å^2^; No. of atoms)		
Protein atoms	22.38 (9552)	21.15 (9589)
FMN atoms	17.03 (124)	–
Malonate atoms	34.08 (77)	31.72 (105)
Water atoms	31.67 (752)	32.66 (757)

Matthews coefficient, the crystal volume per unit of protein molecular weight.

**Figure 4:**
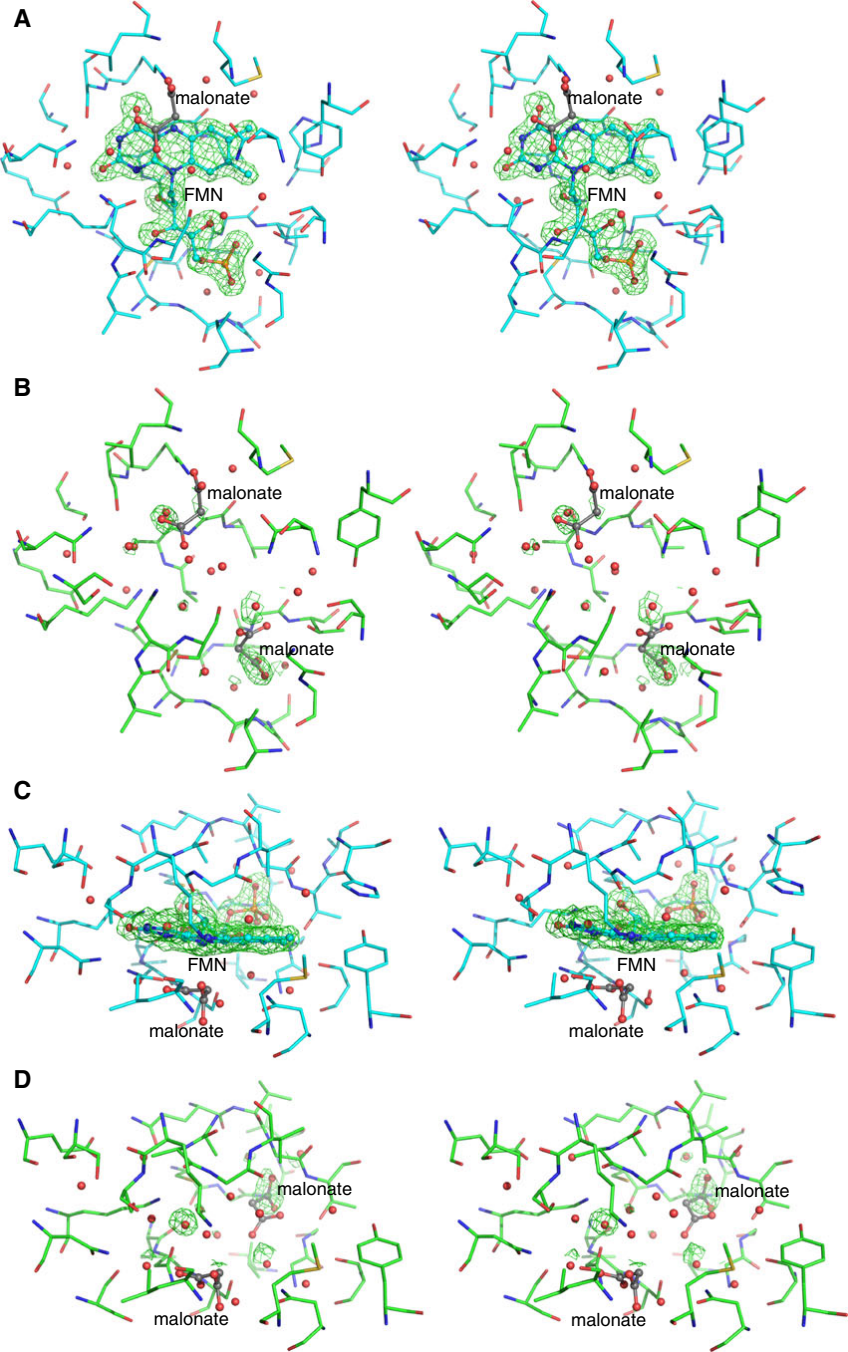
Stereo views of *F*_o_–*F*_c_ omit map of FMN bound to TbDHODH. The contour level is 3.5 sigma. A and B show the FMN‐bound and FMN‐free forms, respectively, of the front view of the plane of the isoalloxazine ring. C and D are the top views of the FMN‐bound and FMN‐free forms, respectively.

*F*_o_–*F*_c_ omit map for FMN at 3.5 sigma (Fig. 4A,C) clearly shows the existence of FMN molecule at the active center of the FMN‐bound form. In contrast, no obvious electron density is observed at the same region in the FMN‐free form (Fig. 4B,D). Shape of the electron density of FMN unambiguously shows a puckering of the isoalloxazine ring of FMN (Fig. 4C). Refinement by Refmac5 program 33 was, therefore, carried out without the flat angle restriction around the N5 and N10 atoms in the relevant parameter file. Puckering angle is 168˚ on average of four chains in the asymmetric unit. Under refinement alternatively with the flat angle restriction, a negative electron density on the *F*_o_–*F*_c_ map was obvious on the hinge (data not shown). Such puckered structures of the isoalloxazine ring of other flavin enzymes have also been reported 34, 35. On the other hand, the flat structure of FMN in DHODH from *T. cruzi* was reported by Inaoka *et al*. 21.

**Figure 5:**
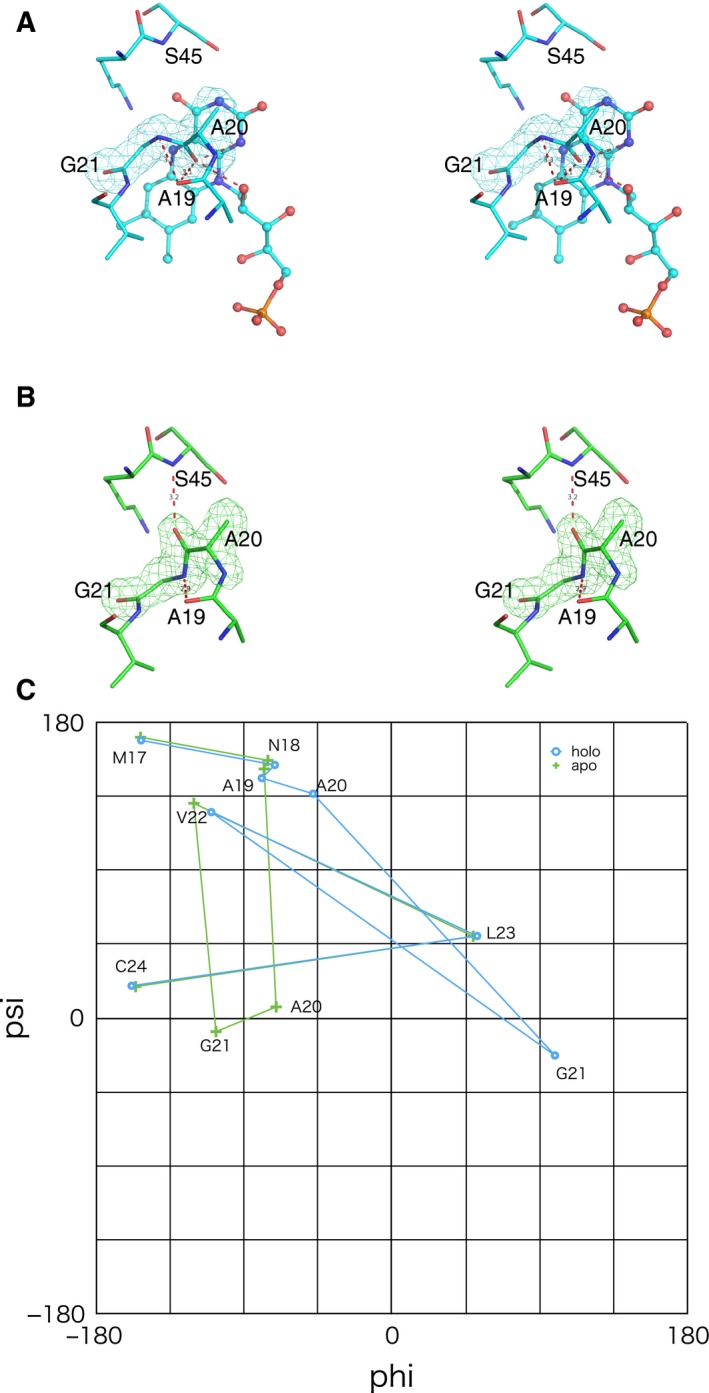
Flipping of peptide plane of TbDHODH between A20 and G21. Stereo views of *F*_o_–*F*_c_ omit map of Ala20‐Gly21 residues in the FMN‐bound (A) and FMN‐free (B) forms. The contour level is 3.5 sigma. (C) Ramachandran plot in region Met17 – Cys24. Cyan (◦) for the FMN‐bound form and green (+) for the FMN‐free form.

In spite of the close similarity in the main‐chain structures of the two forms, we noticed a difference in the peptide bonds between Ala20 and Gly21 (Fig. 5). In the FMN‐bound form, the carbonyl group of Ala20 forms hydrogen bonds with N1 and O2′ atoms of FMN (Fig. 5A). In the FMN‐free form, the peptide plane is flipped into the opposite direction (Fig 5B), where hydrogen bonds are formed between O of Ala20 and N of Ser45 and between N of Gly21 and O of Ala19. This flipping behavior accompanying rearrangements in hydrogen bonds is likely to contribute to stabilizing both the FMN‐free and FMN‐bound forms, which may act as a smooth ‘latch’ for the FMN binding. Ramachandran plot around Ala20–Gly21 (Fig. 5C) indicates that changes in phi‐psi angles are limited within the two residues, without distant perturbation. Thus, the induced fit of the main chain upon FMN binding occurs only in this ‘latch’ region.

**Figure 6:**
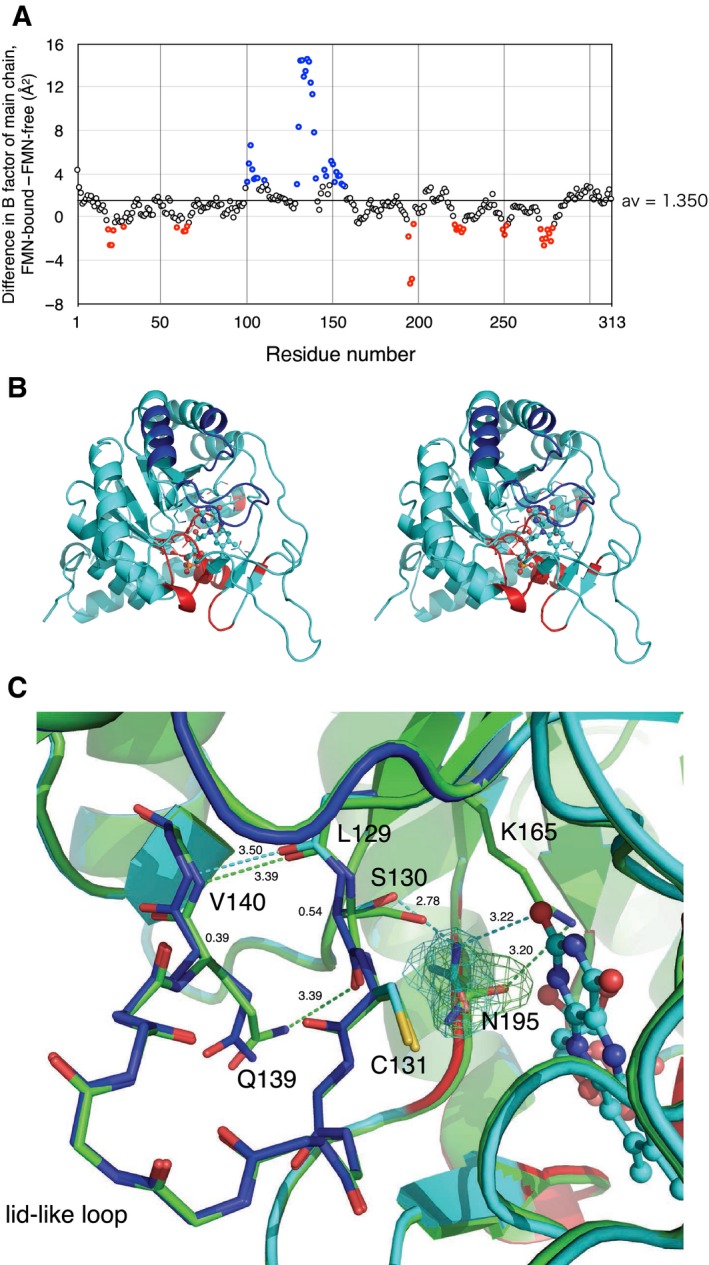
(A) Plot of average B factors of the main chain of TbDHODH. Subtraction of those of the FMN‐free form from those of the FMN‐bound form is plotted versus the residue number. Red and blue symbols represent the highest 30 residues exhibiting negative and positive changes, respectively. (B) Stereographic cartoon model of the monomeric FMN‐bound form additionally colored with blue or red according to panel A. (C) Close up around Asn195. Cartoon models and stick models of some side chains and the main chain of the lid‐like loop of the FMN‐bound form (cyan), which is also colored in the same manner as panel B, are superimposed with that of the FMN‐free form (green). Hydrogen bonds are shown in dashed lines. 2*F*_o_–*F*_c_ maps at 1.0 sigma of Asn195 side chain of the FMN‐free and FMN‐bound forms are also given. FMN is represented in ball‐and‐stick model.

B factors, being related to the positional flexibility, are significantly affected at several regions (Fig. 6A). Average main‐chain B factors of all residues among four chains in asymmetric unit are 17.7 Å^2^ for the FMN‐free form and 18.6 Å^2^ for the FMN‐bound form. The residues with significantly smaller B factors in the FMN‐bound form (red circles in Fig. 6A) appeared particularly around the phosphate and sugar of FMN. Table 2 lists the residues interacting with FMN. Those that interact with the phosphate and sugar typically exhibit smaller B factors in the FMN‐bound form (see difference in B factors without parentheses). In contrast, those interacting with the isoalloxazine ring do not exhibit remarkable decrease, except for Ala20 and Asn195. Similarly for other proteins, the structure around the isoalloxazine ring is kept flexible where a butterfly motion of the ring was allowed 35, 36.

**Table 2:** Polar interactions between FMN and amino acids

FMN	Distance (Å)	Amino acid		
Atom		Atom	Residue	Difference in B factor^a^
O3P	2.90 ± 0.01	N	Gly272	−2.17
	3.43 ± 0.03	N	Thr273	−2.68
O1P	2.79 ± 0.03	N	Gly251	−0.93
	2.75 ± 0.05	N	Gly223	−1.24
O2P	2.82 ± 0.02	N	Thr273	−2.68
	2.73 ± 0.03	OG1		
O3′	3.39 ± 0.01	SG	Cys249	−1.23
	2.94 ± 0.02	O	Val194	−1.92
	3.26 ± 0.05	NZ	Lys165	(−0.70)
O2′	2.83 ± 0.04	O	Ala20	−2.70
	2.93 ± 0.05	NZ	Lys165	(−0.70)
N10	3.37 ± 0.03	O	Ala20	−2.70
N5	3.22 ± 0.09	NZ	Lys44	(0.17)
N1	3.30 ± 0.03	O	Ala20	−2.70
	3.20 ± 0.03	NZ	Lys165	(−0.70)
O4	2.76 ± 0.05	NZ	Lys44	(0.17)
	3.49 ± 0.04	N	Ser45	(0.17)
	3.32 ± 0.04	OG		
N3	2.66 ± 0.03	OG	Ser45	(0.17)
	3.46 ± 0.07	ND2	Asn128	(1.15)
O2	2.93 ± 0.04	NZ	Lys165	(−0.70)
	3.23 ± 0.12	ND2	Asn195	−5.20
	3.12 ± 0.07	ND2	Asn128	(1.15)

a Average B factors among four chains (chain A–D) of the main chain in the FMN‐bound form are subtracted by those of the FMN‐free form. The values exhibiting unremarkable changes that are marked by black symbol in Fig. 6A are shown in parentheses.

On the other hand, residues 100–110 and 130–150 show B factors remarkably increasing upon binding of FMN (blue circles in Fig. 6A). These regions correspond to the lid loop and the helices connecting to the lid (Fig. 6B). The increase is likely to be due to rearrangements in a hydrogen‐bonding network of residues in the loop, which is triggered by interaction between Asn195 and FMN, as follows (Fig. 6C).

In the FMN‐free from, Asn195 side chain is stabilized by contacting with Lys165. Upon binding of FMN, Asn195 side chain forms a hydrogen bond with the isoalloxazine ring, with the carbonate‐amide plane turned around by ~ 90°. This caused a shift of Cα of Ser130 to be more apart (0.54 Å) from Asn195, with forming a hydrogen bond. This shift is conveyed, through the hydrogen bond between Leu129 and Val140, to Gln139 (0.39 Å at Cα of Gln139) at the center of the lid. Consequently, the hydrogen bond between the side chain of Gln139 and the carbonyl group of Ser130 in the FMN‐free form is broken, which make the loop more flexible.

### Binding of FMN to the FMN‐free form analyzed by ITC

**Figure 7:**
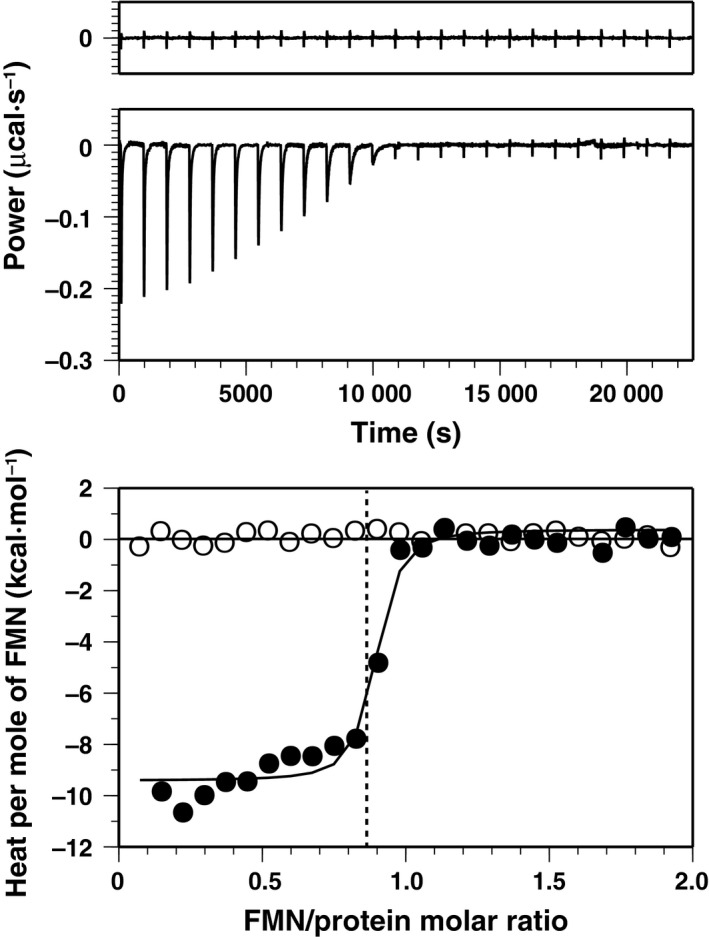
ITC analysis on binding of FMN to the FMN‐free form of TbDHODH. Calorimetric profile for titrations of FMN to the buffer (top) or the protein solution (middle) and derived binding isotherms (bottom) are shown. In the bottom panel, closed and open circles are for titrations to the protein solution and buffer, respectively. A fitting curve to the 1 : 1 binding scheme (see Materials and methods) and the vertical broken line that indicates the number of FMN‐binding sites in the DHODH monomer are also shown.

Binding of FMN to the FMN‐free form was elucidated by ITC (Fig. 7). By fitting of a sigmoidal curve in the binding isotherm, a dissociation constant (*K*_D_) of 1.2 × 10^−8^ m was obtained. The thermodynamic parameters were Δ*H* = −9.3 kcal·mol^−1^, Δ*G* = −10.8 kcal·mol^−1^, and Δ*S* = 4.9 × 10^−3^ kcal·mol^−1^·K^−1^. We could see that the binding reaction is enthalpy‐driven, because contribution of Δ*H* in Δ*G* is as large as 90%. The hydrogen bonds listed in Table 2 are likely to be the major interactions relevant for the enthalpy‐driven mechanism. It was reported that concentration of FMN in mitochondria was estimated to be several micromolar 37, which is sufficient enough to constantly occupy the pocket by FMN, considering the above dissociation constant.

## Discussion

The flipping of the peptide plane between Ala20 and Gly21 is concomitant with the rearrangement of hydrogen bonds, which was considered a ‘latch’ for binding of FMN; this stabilizes both the forms and reduces the energy difference. This kind of flipping was often found in β‐turn region, of which the energy barrier was estimated to be 3 kcal·mol^−1^ 38; this is smaller than the Δ*G* value of FMN binding in the absolute value, but not negligible. Latch‐like peptide flipping was reported for flavodoxin from *Clostridium beijerinckii* upon one‐electron reduction in FMN 39, 40; the resultant semiquinone FMN was stabilized by hydrogen bonding with the flipped carbonyl oxygen. Similar peptide flipping has been observed in the ligand‐binding region of 6‐oxopurine phosphoribosyltransferase 41. In this case, open and closed forms with regard to the catalytic loop were concomitant with the peptide flipping. Similar to the present study, the carbonyl oxygen of the residue before Gly kept hydrogen bonding with different residues in the two forms.

Changes in flexibility of the lid part upon the orotate binding have been observed by crystallography 19, 20; the lid part was found stabilized in a closed conformation in the orotate‐bound form, while the lid was found in an open conformation or in disordered in the substrate‐free form. However, no regulatory mechanism to control the flexibility has been hypothesized. We raised a possibility that the hydrogen‐bond network of Ser130, Asn195, and the isoalloxazine ring of FMN is a key structure to control the flexibility of the lid. Together with the mechanism that bending of the isoalloxazine ring is affected by the redox state and/or the presence of the substrates under π‐electron stacking with the ring 36, we suggest that the substrate binding on the isoalloxazine ring of FMN influences the flexibility in the lid part as mediated by the same hydrogen‐bond network. It should be noted that release of orotate was the rate‐limiting step of the whole enzyme reaction 32.

No obvious induced fit‐type conformational change upon FMN binding was found, except for the latch‐like local flipping. Oppositely, increased flexibility of the lid in the FMN‐bound form was signified by comparison of B factors. These changes upon FMN binding observed in DHODH are different from the cases with other FMN‐binding proteins. For example, induced fit‐type conformational changes of the isoalloxazine‐binding loops and some residues around the FMN‐binding site were found for flavodoxins from plant and *H. pylori* 25, 26. Also, an NMR study revealed that flavodoxins showed motions on the μs‐ms timescale around the FMN‐binding site in the FMN‐free form, which largely disappeared in the FMN‐bound form 27. Moreover, an NMR H/D exchange experiment on *A. vinelandii* flavodoxin showed that binding of FMN decreased opening motions of residues spread over the whole protein 28. Namely, in flavodoxin, binding of FMN has tendency to fix the structure around the FMN‐binding site, as well as the whole protein. The FMN‐free form of dibenzothiophene monooxygenase DszC from *R. erythropolis* had the same structure to that of the FMN‐bound form, except for a loop region near the substrate channel, where the loop in the FMN‐free form is open for substrate to access the reaction pocket 29; DszC is a two‐component monooxygenase that binds FMN as a substrate but not as a prosthetic group. The fact that the FMN‐free form is in an open form is therefore reasonable. Contrary to DszC, the lid part of the FMN‐free form of DHODH is in a closed form as well as that of the FMN‐bound form.

We demonstrated here that the FMN‐free form of *T. brucei* DHODH is stable as a correctly folded ‘native’ protein. Therefore, we suggest that this form can be also considered as a target for drug discovery; the large space of FMN‐binding pocket may be useful for development of high‐affinity compounds. If these compounds compete out FMN, they should work as strong inhibitors. Although concentration of FMN in cells of the parasitic protozoan is not available, it was estimated to be 0.5–6.0 amol·cell^−1^ for mammalian cells 42, equivalent to 2.5–30 μm, when the cell volume is assumed to be 200 μm^3^. Considering that the values include FMN bound to other proteins noncovalently, concentration of free FMN may be significantly smaller. With the dissociation constant of 12 nm obtained by ITC, it would be possible that the FMN‐free form partially exists in the *T. brucei* cell. Therefore, if we succeed in developing compounds that compete out FMN *in vitro*, it is worth examining whether they penetrate into the cell and truly behave as inhibitors *in situ*.

## Materials and methods

### Enzyme expression and purification

The codon‐optimized synthesized gene that coded for the A115V mutant of DHODH of *T. brucei brucei* strain 927/4 GUTat10.1 (GeneDB Tb927.5.3830) was purchased from Thermo Fisher Scientific Inc. This sequence was identical to that of PDB: 2B4G entry 20. The gene was incorporated between the *Sma*I and *Not*I sites of expression vector pET‐47b (Merck Millipore, Darmstadt, Germany). The obtained vector was designed to code for DHODH with a His tag and the cleavage sequence of human rhinovirus (HRV) 3C protease, that is, MetAla(His)_6_SerAlaAlaLeuGluValLeuPheGln↓GlyProGlySer (arrow indicates the cleavage site), at the N terminus. Protein was expressed by *E. coli* strain BL21(DE3) in LB or TB medium with 1 mm isopropyl β‐d‐1‐thiogalactopyranoside at 25 °C for 6 h. Harvested cells were lysed and homogenized by sonication in a lysis buffer, 50 mm sodium phosphate (pH 8.0), 300–500 mm NaCl, and 1 mm PMSF. The lysis buffer was supplemented with 10 mm imidazole, 10 μg·mL^−1^ RNaseA, 5 μg·mL^−1^ DNaseI, 0.025 tablet·mL^−1^ protease inhibitor cocktail, and 520 kU lysozyme, for the FMN‐reconstitution assay.

Supernatant of centrifugation was applied to Ni‐NTA column (Qiagen, Hilden, Germany) equilibrated in 50 mm sodium phosphate (pH 8.0), 300–500 mm NaCl, 10–20 mm imidazole, and 1 mm PMSF. Proteins were eluted by the above buffer containing 250 mm imidazole. After removing imidazole, protein was digested by HRV 3C protease (Merck Millipore or Wako, Osaka, Japan) of a 1 (Unit):50–100 (μg protein) ratio at 4 °C. Undigested protein and HRV 3C protease were trapped by Ni‐NTA column. Unbound fraction was collected and desalted with an ultrafiltration. The sample was applied to RESOURCE Q (GE Healthcare, Buckinghamshire, UK) anion‐exchange column chromatography equilibrated at 50 mm Tris/HCl (pH 8.0), to separate the FMN‐free form from the FMN‐bound form. Adsorbed proteins were eluted with a gradient of 0–500 mm NaCl for 20 column volumes. Elution pattern was monitored at 450 nm and 280 nm. For crystallization, each fraction was further purified with Superdex 200 (GE Healthcare) gel filtration in 10 mm Tris/HCl (pH 8.0) and 150 mm NaCl.

### Enzyme assay

Concentration of the FMN‐free form was evaluated by absorbance at 280 nm using an extinction coefficient 16 390 m ^−1^·cm^−1^, as calculated from amino acid content 43. Concentration of the FMN‐bound form was evaluated by Bio‐Rad DC Protein Assay (Hercules, CA, USA), by use of the FMN‐free form as a standard calibrated with the absorbance, yielding extinction coefficients of 45 700 and 9910 m ^−1^·cm^−1^ at 280 nm and 450 nm, respectively for the FMN‐bound form.

Dihydroorotate dehydrogenase activity was assayed as produced orotate by measuring absorption at 300 nm (ε = 2.65 mm ^−1^·cm^−1^) 44. Reaction was started by adding 50 nm (final concentration) DHODH into a reaction mixture containing 100 mm Tris/HCl (pH 8.0), 1 mm sodium fumarate, and 0.5 mm dihydroorotate. Volume was 2 mL in an optical cell. Absorbance change was traced by Beckman Coulter DU640 spectrophotometer. Temperature of the cell holder was controlled at 25 °C.

### Reconstitution of FMN

A 2.8 μL of 35 μm FMN‐free DHODH in 10 mm Tris/HCl (pH8.0) and 150 mm NaCl was mixed with the equal volume of 5 mm FMN solution. After incubation for 1 min at 4 °C, the mixture was diluted into 1 mL of 100 mm Tris/HCl (pH8.0) and immediately used as an enzyme source.

### Thermal denaturation assay

Fluorescence‐based assay was performed by a StepOnePlus™ real‐time PCR system (Thermo Fisher, Waltham, MA, USA). Proteins of 0.2 mg·mL^−1^ were dissolved in 10 mm HEPES (pH 7.5), 150 mm NaCl, 0.005% Tween 20, and 0.1% SYPRO™ Orange Protein Gel Stain solution (Thermo Fisher). Temperature was raised from 25 to 85 °C by a step of 0.5 °C. Melting temperatures were calculated by stepone software ver. 2.3 (Thermo Fisher). The experiments were triplicated.

### Crystallization and X‐ray crystallography

The purified FMN‐free and FMN‐bound forms were crystallized by the hanging‐drop vapor diffusion method. Protein drop at 10 mg·mL^−1^ in 10 mm Tris/HCl (pH 8.0) was mixed with the equal volume of a reservoir solution, 1.5–2.5 m sodium malonate (pH 6.0) and 0.1 m sodium citrate (pH 5.6). Crystals grew at 20 °C for 1–3 weeks, which were frozen up with liquid nitrogen without any additional cryoprotectant.

X‐ray diffraction data were collected at NE3A beam line of Photon Factory in KEK, Japan, and processed by program HKL2000 45. Program Phaser 46 in CCP4 suits 47 was used for phase determination by molecular replacement with *T. brucei* DHODH (PDB: 2B4G) 20. Model building and refinement were carried out by programs COOT 48 and REFMAC5 33, respectively. The planner angle restrictions of N1 and N10 atoms were removed from the parameter file, FMN.cif, for REFMAC5 refinement. Crystallographic statistics are given in Table 1. Structural figures were rendered by program pymol 49. The coordinates and diffraction data for the FMN‐free form (5XFW) and the FMN‐bound form (5XFV) have been deposited in the Protein Data Bank.

### ITC analysis

Binding of FMN to the FMN‐free DHODH was analyzed using a VP‐ITC calorimeter (GE Healthcare) at 298 K. To the reaction cell of 1427 μL containing the protein of 9.5 μm dissolved in a buffer [20 mm sodium phosphate (pH 8.0) containing 150 mm NaCl], successive injections of 5 μL each from the syringe containing the FMN solution (0.2 mm FMN in the same buffer) were carried out after degassing the solutions. A reference experiment injecting the FMN solution to the buffer in the cell was also performed. A fitting analysis of data was performed by in‐house Fortran 90 programs calling imsl 6.0 subroutines (Visual Numerics, Houston, TX, USA), essentially according to equations described in the vendor's manual, with a correction of concentration 50. Briefly, the 1 : 1 binding scheme was introduced as

(1) K=Θ/(1−Θ)[FMN]

where *K* is the binding constant, that is, the reciprocal of *K*_D_, Θ is fraction of the binding sites in the protein occupied by FMN, and [FMN] is concentration of free FMN. The number of the binding sites in the protein (*n*) is not necessarily 1.0 in the data fitting, that is,

(2) $$X_{t}=\left[\text{FMN}\right]+n\Theta M_{t}$$

where *X*_t_ and *M*_t_ are bulk concentrations of FMN and the protein, respectively. Thermodynamic parameters were obtained by analysis of the binding isotherm, that is, relation between heat observed by a shot of titration and the molar ratio of bulk concentrations of FMN and protein.

## Authors contributions

TK, OT, TY, and KY carried out the experiments. TK, KY, and OT wrote the manuscript. KF, KY, IN, and HS designed the project. All authors reviewed the manuscript.
